# Bioinformatics analysis to identify the key genes affecting the progression and prognosis of hepatocellular carcinoma

**DOI:** 10.1042/BSR20181845

**Published:** 2019-02-22

**Authors:** Yingai Zhang, Shunlan Wang, Jingchuan Xiao, Hailong Zhou

**Affiliations:** 1 Institute of Tropical Agriculture and Forestry, Hainan University, Haikou 570228, China; 2 Central Laboratory, Central South University Xiangya School of Medicine Affiliated Haikou Hospital, Haikou 570208, China; 3 State Key Laboratory of Marine Resource Utilization in South China Sea, Hainan University, Haikou 570228, China

**Keywords:** differentially expressed genes, hepatocellular carcinoma, protein-coding genes, protein-protein interaction WORK, survival analysis

## Abstract

Hepatocellular carcinoma (HCC) is the most frequent primary liver cancer, which has poor outcome. The present study aimed to investigate the key genes implicated in the progression and prognosis of HCC. The RNA-sequencing data of HCC was extracted from The Cancer Genome Atlas (TCGA) database. Using the R package (DESeq), the differentially expressed genes (DEGs) were analyzed. Based on the Cluepedia plug-in in Cytoscape software, enrichment analysis for the protein-coding genes amongst the DEGs was conducted. Subsequently, protein–protein interaction (PPI) network was built by Cytoscape software. Using survival package, the genes that could distinguish the survival differences of the HCC samples were explored. Moreover, quantitative real-time reverse transcription-PCR (qRT-PCR) experiments were used to detect the expression of key genes. There were 2193 DEGs in HCC samples. For the protein-coding genes amongst the DEGs, multiple functional terms and pathways were enriched. In the PPI network, cyclin-dependent kinase 1 (CDK1), polo-like kinase 1 (PLK1), Fos proto-oncogene, AP-1 transcription factor subunit (FOS), serum amyloid A1 (SAA1), and lysophosphatidic acid receptor 3 (LPAR3) were hub nodes. CDK1 interacting with PLK1 and FOS, and LPAR3 interacting with FOS and SAA1 were found in the PPI network. Amongst the 40 network modules, 4 modules were with scores not less than 10. Survival analysis showed that anterior gradient 2 (*AGR2*) and *RLN3* could differentiate the high- and low-risk groups, which were confirmed by qRT-PCR. *CDK1, PLK1, FOS, SAA1*, and *LPAR3* might be key genes affecting the progression of HCC. Besides, *AGR2* and *RLN3* might be implicated in the prognosis of HCC.

## Background

Hepatocellular carcinoma (HCC) is the most frequent primary liver cancer and causes the most deaths in cirrhosis patients [1]. HCC usually occurs in people with chronic liver inflammation, which is closely related to virus infection or alcohol and aflatoxin exposure [2]. Epidemiological data show that the main risk factors for HCC are as follows: (i) Hepatitis B or hepatitis C virus infection. (ii) Aflatoxin. (iii) Drinking wastewater or pond water containing a large amount of organochlorine compounds and algae toxins. (iv) Other factors such as family aggregation, selenium deficiency, alcoholic and nutritional cirrhosis [2,3]. The therapeutic schedules of HCC depend on disease stage, operative tolerance, and possibility of liver transplant [3,4]. The outcome of HCC patients is usually poor, because 80–90% HCCs cannot be resected completely and leads to death in 3–6 months [5,6]. HCC is amongst the most common tumors and results in more than 670000 deaths per year globally [7]. Therefore, the mechanisms of HCC needed to be explored to improve its therapies.

A lot of studies indicated that Forkhead box C1 (*FoxC1*), αB-crystallin, Zinc finger and BTB domain containing 20 (*ZBTB20*), Dysregulated B-cell translocation gene 1 (*BTG1*), Homeobox A13b (*HOXA13*), DEK proto-oncogene (*DEK*), Ubiquitin-specific protease 7 (*USP7*), and Acyl-CoA Ligase 4 (*ACSL4*) played important roles in the occurrence and progression of HCC, and they may serve as novel prognostic factors and therapeutic targets for HCC [8–18]. *FoxC1* may facilitate HCC metastasis via inducing epithelial–mesenchymal transition (EMT) and up-regulating neural precursor cell expressed, developmentally down-regulated 9 (*NEDD9*) [8,9]. The expression of αB-crystallin has correlations with the invasion and metastasis of HCC cells [10]. *ZBTB20* expression was up-regulated in HCC and related to adverse prognosis in HCC patients [11,12]. *BTG1* may be involved in hepatocarcinogenesis and may be taken as a biomarker for the carcinogenesis and progression of HCC [13]. *HOXA13* may affect angiogenesis, progression, and outcome of HCC, and serum *HOXA13* may be utilized for early diagnosis and prognosis prediction of HCC patients [14]. *DEK* is reported to be implicated in hepatocyte differentiation and acts as a candidate marker for the prognosis and staging of HCC [15,16]. *USP7* and *ACSL4* are up-regulated in HCC samples, which are related to a poor survival [17,18]. Despite these findings, the key genes having influences on the progression and prognosis of HCC patients have not been comprehensively revealed.

Various *in silico* tools of bioinformatics are widely applied for assessing gene expression levels and screening outstanding genes from RNA-sequencing data and next-generation sequencing data and their possible implication in growth of different types of cancer [19]. In the present study, differential expression analysis, enrichment analysis, network analysis, and survival analysis successively were conducted to find out the key genes that affected the prognosis of HCC. In addition, quantitative real-time reverse transcription-PCR (qRT-PCR) experiments were conducted to confirm the expression of key genes. The present study might help to predict the outcome of HCC patients and develop novel therapies.

## Materials and methods

### Microarray data

The RNA-sequencing data of HCC was obtained from The Cancer Genome Atlas (TCGA, https://cancergenome.nih.gov/) database, which was based on the platform of llumina HiSeq 2000 RNA Sequencing. The TCGA dataset contained 419 samples, including 369 HCC samples and 50 normal control samples [20]. Meanwhile, the corresponding clinical data of the HCC samples were downloaded from the TCGA database.

### Data preprocessing and differential expression analysis

The genes with counts per million (cpm) < 1 in more than 10% samples were taken as low expressed genes and removed. According to the annotation files in GENCODE database (version 22) [21], ensemble gene IDs (ENSGs) were mapped to gene symbols and gene types (coding genes or non-coding genes). Using the R package DESeq (http://www.bioconductor.org/packages/release/bioc/html/DESeq.html) [22], the differentially expressed genes (DEGs) between HCC samples and normal samples were analyzed. The thresholds for differential expression analysis were set as |log fold-change (FC)| ≥ 2 and false discovery rate (FDR) < 0.01.

### Functional and pathway enrichment analyses

Gene Ontology (GO) database introduces the biological process (BP), cellular component (CC), and molecular function (MF) for proteins [23]. Kyoto Encyclopedia of Genes and Genomes (KEGG) database included the functions of genes and can be utilized for the functional prediction of gene lists [24]. Using Cluepedia plug-in (http://apps.cytoscape.org/apps/cluepedia) [25] in Cytoscape software, the protein-coding genes amongst the DEGs were performed with GO and KEGG enrichment analyses. The terms with FDR < 0.05 were significant results. Besides, the significant KEGG pathways were presented by the R package pathview (http://r-forge.r-project.org/projects/pathview/) [26].

### Protein–protein interaction network analysis

Protein–protein interaction (PPI) pairs were predicted for the protein-coding genes amongst the DEGs using the STRING (http://www.string-db.org/) [27] database, with the combined score set as 700. Afterward, the PPI network was visualized using Cytoscape software (http://www.cytoscape.org) [28]. Moreover, the MCODE plug-in (http://apps.cytoscape.org/apps/MCODE) [29] in Cytoscape software was used for identifying the significant modules in PPI network.

### Patient samples

Clinical samples were collected from 26 HCC patients who underwent surgery at the Central South University Xiangya School of Medicine Affiliated Haikou Hospital from 2013 to 2017. Meanwhile the adjacent non-tumor liver tissues from the HCC patients were obtained as the normal controls. None of the patients accepted radiation therapy and/or chemotherapy before surgery and all of them have signed the informed consent. Ethical approval for the study was provided by the ethics committee of Central South University Xiangya School of Medicine Affiliated Haikou Hospital, and the research has been carried out in accordance with the World Medical Association Declaration of Helsinki.

### qRT-PCR analysis

The total RNAs of the samples were extracted using TRIzol (Thermo Fisher Scientific, Waltham, MA, U.S.A.), detected by UV absorbance (A_260/280_) and agarose gel. qRT-PCR was carried out in the ABI Prism 7500 PCR system (Applied Biosystems, Foster City, CA, U.S.A.) following the standard instructions. GADPH acted as the internal criterion for the targetted genes. 2^−ΔΔ*c*^_t_ method was taken to calculate the relative expressions of the targetted genes. The primers’ sequences for GADPH were (F) 5′-GCACCGTCAAGGCTGAGAAC-3′ and (R) 5′-GCCTTCTCCATGGTGGTGAA-3′. PCR was performed in three parallel holes.

### Survival analysis

Based on univariate COX regression analysis [30], the correlations between the DEG and overall survival (OS) were analyzed. Combined with the R package survival (https://CRAN.R-project.org/view=Survival) [31], the HCC samples were divided into high- and low-risk groups and then the OS differences between the two groups were analyzed using Kaplan–Meier (KM) method [32]. Student’s *t* tests and one-way ANOVAs were used in either two or multiple groups for statistical significance, with *P*<0.05 considered significant.

## Results

### Differential expression analysis

Relative to normal samples, a total of 2193 DEGs (including 1964 up-regulated genes and 229 down-regulated genes) in HCC samples were screened. Amongst these DEGs, there were 1800 protein-coding genes and 232 long non-coding RNAs (lncRNAs).

### Functional and pathway enrichment analyses

**Figure 1:**
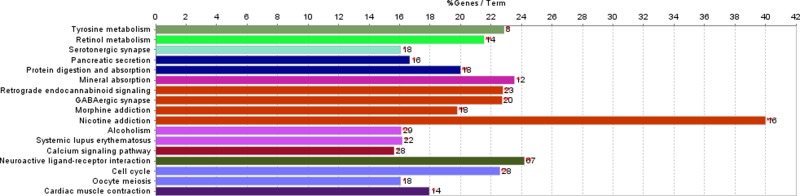
The pathways enriched for the protein-coding genes amongst the DEGs

Enrichment analysis showed that multiple GO terms and 17 KEGG pathways were enriched for the protein-coding genes amongst the DEGs. The obtained GO terms mainly were multicellular organismal development (GO_BP, FDR = 1.09E-20), neurone part (GO_CC, FDR = 1.19E-08), and ion channel activity (GO_MF, FDR = 1.25E-10) (Table 1). Besides, the enriched pathways mainly included neuroactive ligand–receptor interaction (FDR = 7.13E-14), nicotine addiction (FDR = 2.62E-06), and cell cycle (FDR = 4.10E-05) (Figure 1).

**Table 1:** The top five GO terms enriched for the protein-coding genes amongst the DEGs

Category	Description	Count	FDR
GO_BP	Multicellular organismal development	645	1.09E-20
GO_BP	System development	581	1.29E-19
GO_BP	Cell–cell signaling	217	2.10E-16
GO_BP	Organ development	431	2.27E-16
GO_BP	Synaptic transmission	145	5.63E-15
GO_CC	Neurone part	189	1.19E-08
GO_CC	Ion channel complex	62	1.87E-08
GO_CC	Extracellular space	203	2.22E-08
GO_CC	Integral component of plasma membrane	228	7.65E-08
GO_CC	Transmembrane transporter complex	65	9.23E-08
GO_MF	Ion channel activity	83	1.25E-10
GO_MF	Sequence-specific DNA binding	167	1.38E-10
GO_MF	Gated channel activity	71	1.43E-10
GO_MF	Substrate-specific channel activity	86	1.45E-10
GO_MF	Channel activity	89	1.45E-10

### PPI network analysis

**Figure 2:**
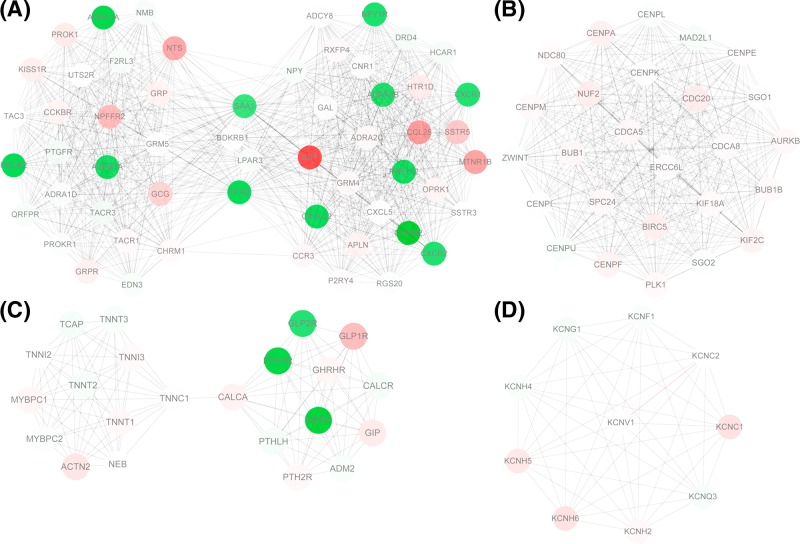
PPI network analysis The module 1 (**A**), module 2 (**B**), module 3 (**C**), and module 4 (**D**) identified from the PPI network. Red and green circles represent up-regulated and down-regulated genes, respectively.

The PPI network for the protein-coding genes amongst the DEGs was constructed, which had 926 nodes and 3748 edges. The degrees of network nodes obeyed exponential distribution (r-squared = 0.849), therefore, the PPI network was a scale-free network and some network nodes had higher degrees. According to node degrees, cyclin-dependent kinase 1 (CDK1, degree = 76), polo-like kinase 1 (PLK1, degree = 72), Fos proto-oncogene, AP-1 transcription factor subunit (FOS, degree = 63), serum amyloid A1 (SAA1, degree = 59), and lysophosphatidic acid receptor 3 (LPAR3, degree = 59) were the top five nodes. Especially, CDK1 had interactions with both PLK1 and FOS, and LPAR3 could interact with FOS and SAA1 in the PPI network. Besides, FOS also had interaction with relaxin 3 (*RLN3*) in the network. Furthermore, a total of 40 network modules were identified. The modules with scores no less than 10 were presented in Figure 2, including module 1 (score = 32.036, involving 56 nodes and 881 edges), module 2 (score = 26, involving 26 nodes and 325 edges), module 3 (score = 10.571, involving 22 nodes and 111 edges), and module 4 (score = 10, involving 10 nodes and 45 edges) (Table 2).

**Table 2:** The 12 genes that could differentiate the survival differences of HCC samples

Gene	*P*-value
*AGR2*	0.024
*CRISP2*	0.04
*IL31RA*	0.0044
*KCNC2*	0.026
*LINC01477*	0.013
*LOC105372556*	0.023
*PLVAP*	0.011
*RLN3*	0.043
*SOX14*	0.011
*TEDDM1*	0.023
*VWA5B2*	0.048
*ZNF280A*	0.016

Abbreviations: AGR2, anterior gradient 2; CRISP2, cysteine-rich secretory protein 2; IL31RA, interleukin 31 receptor A; KCNC2, potassium voltage-gated channel subfamily C member 2; LINC01477, long intergenic non-protein coding RNA 1477; PLVAP, plasmalemma vesicle-associated protein; SOX14, SRY-box 14; TEDDM1, transmembrane epididymal protein 1; VWA5B2, von Willebrand factor A domain containing 5B2.

### Survival analysis

**Figure 3:**
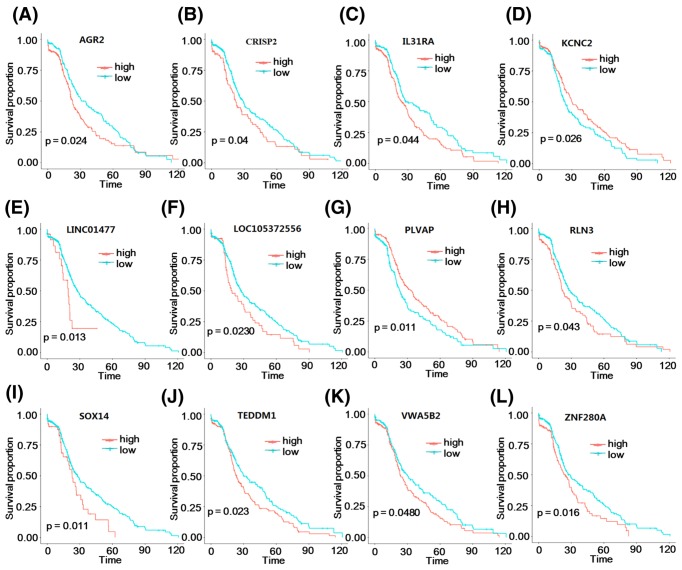
Survival analysis The KM survival curves for *AGR2* (**A**), *CRISP2* (**B**), *IL31RA* (**C**), *KCNC2* (**D**), *LINC01477* (**E**), *LOC105372556* (**F**), *PLVAP* (**G**), *RLN3* (**H**), *SOX14* (**I**), *TEDDM1* (**J**), *VWA5B2* (**K**), and *ZNF280A* (**L**). Red and blue separately represent high- and low-risk groups.

Based on univariate COX regression analysis, a total of 116 DEGs were found to be correlated with the OS of HCC samples (*P*-value <0.05). According to the median value of gene expression, the HCC samples were divided into high- and low-risk groups. Subsequently, the OS differences between the two groups were analyzed. KM survival curves showed that 12 genes (including anterior gradient 2, *AGR2*; cysteine-rich secretory protein 2, *CRISP2*; interleukin 31 receptor A, *IL31RA*; long intergenic non-protein coding RNA 1477, *LINC01477*; *RLN3*; SRY-box 14, *SOX14*; transmembrane epididymal protein 1, *TEDDM1*; von Willebrand factor A domain containing 5B2, *VWA5B2*; zinc finger protein 280A, *ZNF280A*; potassium voltage-gated channel subfamily C member 2, *KCNC2*; *LOC105372556*; and plasmalemma vesicle-associated protein, *PLVAP*) could divide the HCC samples into two groups that had OS differences (*P*-value <0.05) (Figure 3).

### Expressions of genes differentiated the survival differences in HCC in clinical samples

**Figure 4:**
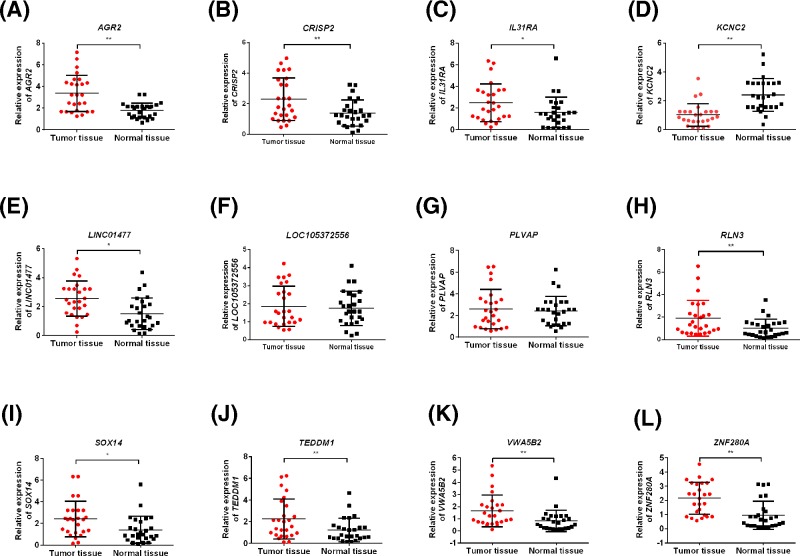
Expressions of genes differentiated the survival differences in HCC in clinical samples The expressions of *AGR2* (**A**), *CRISP2* (**B**), *IL31RA* (**C**), *KCNC2* (**D**), *LINC01477* (**E**), *LOC105372556* (**F**), *PLVAP* (**G**), *RLN3* (**H**), *SOX14* (**I**), *TEDDM1* (**J**), *VWA5B2* (**K**), and *ZNF280A* (**L**) in HCC tissues and adjacent normal tissues. Data were shown as mean ± S.D., *n*=26. *P<0.05 and **P<0.01.

We, then, examined the expressions of genes differentiating the survival differences of HCC in HCC tissues and adjacent normal tissues by qRT-PCR. As shown in Figure 4, the expressions of *AGR2, CRISP2, IL31RA, LINC01477, RLN3, SOX14, TEDDM1, VWA5B2*, and *ZNF280A* were significantly increased in HCC tissues compared with the normal tissues (*P*-value <0.05) (Figure 4), which were consistent with the results of differential expression analysis.

## Discussion

In the present study, a total of 2193 DEGs (including 1800 protein-coding genes and 232 lncRNAs) in HCC samples were identified. For the protein-coding genes amongst the DEGs, functional and pathway enrichment analyses were carried out. In the PPI network, CDK1 (degree = 76), PLK1 (degree = 72), FOS (degree = 63), SAA1 (degree = 59), and LPAR3 (degree = 59) were key nodes. A total of 40 network modules were identified, amongst which 4 modules were with scores no less than 10. Survival analysis suggested that 12 genes (including *AGR2* and *RLN3*) could divide the HCC samples into high- and low-risk groups. Furthermore, the expressions of nine genes (including *AGR2* and *RLN3*) were confirmed by qRT-PCR experiments.

*CDK1* interacts with apoptin during HCC tumorigenesis, and their link may play a role in mediating tumor cell apoptosis [33]. Via directly suppressing *CDK1* and v-akt murine thymoma viral oncogene homolog 3 (*AKT3*) expression and indirectly inhibiting cyclinD1 expression; *miR-582-5p* functions in the development and progression of HCC [34]. *PLK1* expression is significantly up-regulated in HCC tissues, therefore, *PLK1* may be a potential marker for the prognosis of HCC and targetting *PLK1* may be applied for the diagnosis and therapy of the disease [35,36]. *PLK1* is overexpressed in HCC samples relative to normal controls, and its knockdown can induce apoptosis of tumor cells via the endonuclease-G pathway [37]. *FOS* expression is inhibited by *miR-139* down-expression in HCC cells with high metastatic potency, which promotes the metastasis in HCC [38]. Through suppressing the expression of the oncogene FOS, dysregulated *miR-101* is implicated in the pathogenesis of HCC [39]. CDK1 had interactions with both PLK1 and FOS in the PPI network, suggesting that *CDK1* might be involved in the development and progression of HCC through interacting with *PLK1* and *FOS*.

The preoperative serum level of *SAA* is closely correlated with tumor size and tumor stage, implicating that *SAA* overexpression can serve as a promising prognostic factor for HCC patients [40,41]. The *LPAR1/LPAR3* expression is increased in hepatoma cell line SKHep1, and the LPA-LPAR3 signaling may play an essential role in tumor invasiveness/expansion [42]. Several *LPAR* subtypes are detected in HCC samples, and the suppression of LPA-LPAR signaling represses the motility and proliferation of HCC cells [43]. LPAR3 could also interact with FOS and SAA1 in the PPI network, indicating that *LPAR3* might function in pathogenesis of HCC via interacting with *FOS* and *SAA1*.

High *AGR2* expression level contributes to the metastasis of HCC cells through acting on mitogen-activated protein kinase (MAPK) and Caspase pathway, which results in the unfavorable prognosis of HCC patients [44]. *AGR2* overexpression is found in various tumors including fibrolamellar HCC, and the dysregulated *AGR2* is a phenotypic characteristic of cholangiocytes [45,46]. *RLN2* expression is found to be up-regulated in HCC tissues, which can be taken as a predictor for tumor progression and adverse prognosis [47]. Therefore, *AGR2* and *RLN3* might be correlated to the prognosis of HCC patients.

## Conclusion

In conclusion, a total of 2193 DEGs in HCC samples were identified. Besides, *CDK1, PLK1, FOS, SAA1, LPAR3, AGR2*, and *RLN3* might play important roles in the progression and prognosis of HCC. Nevertheless, lacking experiments was main limitation of the present study and more experiments should be designed to support our results.

## Abbreviations

ACSL4: acyl-CoA ligase 4
AGR2: anterior gradient 2
BP: biological process
BTG1: B-cell translocation gene 1
CC: cellular component
CDK1: cyclin-dependent kinase 1
CRISP2: cysteine-rich secretory protein 2
DEG: differentially expressed gene
FDR: false discovery rate
FOS: Fos proto-oncogene, AP-1 transcription factor subunit
FoxC1: Forkhead box C1
GO: gene ontology
HCC: hepatocellular carcinoma
HOXA13: homeobox A13b
KEGG: Kyoto Encyclopedia of Genes and Genomes
KM: Kaplan–Meier
LINC01477: long intergenic non-protein coding RNA 1477
lncRNA: long non-coding RNA
LPAR3: lysophosphatidic acid receptor 3
OS: overall survival
PLK1: polo-like kinase 1
PPI: protein–protein interaction
qRT-PCR: quantitative real-time reverse transcription-PCR
RLN3: relaxin 3
SAA: serum amyloid A1
SOX14: SRY-box 14
TCGA: The Cancer Genome Atlas
TEDDM1: transmembrane epididymal protein 1
USP7: ubiquitin-specific protease 7
VWA5B2: von Willebrand factor A domain containing 5B2
ZBTB20: zinc finger and BTB domain containing 20
ZNF280A: zinc finger protein 280A
